# Quinolin-6-Yloxyacetamides Are Microtubule Destabilizing Agents That Bind to the Colchicine Site of Tubulin

**DOI:** 10.3390/ijms18071336

**Published:** 2017-06-22

**Authors:** Ashwani Sharma, Gonzalo Sáez-Calvo, Natacha Olieric, Francisco de Asís Balaguer, Isabel Barasoain, Clemens Lamberth, J. Fernando Díaz, Michel O. Steinmetz

**Affiliations:** 1 Laboratory of Biomolecular Research, Division of Biology and Chemistry, Paul Scherrer Institut, CH-5232 Villigen, Switzerland; ashwani.sharma@psi.ch (A.S.); natacha.olieric@psi.ch (N.O.); 2 Centro de Investigaciones Biológicas, Consejo Superior de Investigaciones Científicas, Ramiro de Maeztu 9, 28040 Madrid, Spain; g.saez.calvo@gmail.com (G.S.-C.); pacobal@cib.csic.es (F.d.A.B.); i.barasoain@cib.csic.es (I.B.); 3 Chemical Research, Syngenta Crop Protection AG, Schaffhauserstrasse 101, CH-4332 Stein, Switzerland; clemens.lamberth@syngenta.com

**Keywords:** microtubules, microtubule targeting agents, quinolin-6-yloxyacetamides, multidrug resistance

## Abstract

Quinolin-6-yloxyacetamides (QAs) are a chemical class of tubulin polymerization inhibitors that were initially identified as fungicides. Here, we report that QAs are potent anti-proliferative agents against human cancer cells including ones that are drug-resistant. QAs act by disrupting the microtubule cytoskeleton and by causing severe mitotic defects. We further demonstrate that QAs inhibit tubulin polymerization in vitro. The high resolution crystal structure of the tubulin-QA complex revealed that QAs bind to the colchicine site on tubulin, which is targeted by microtubule-destabilizing agents such as colchicine and nocodazole. Together, our data establish QAs as colchicine-site ligands and explain the molecular mechanism of microtubule destabilization by this class of compounds. They further extend our structural knowledge on antitubulin agents and thus should aid in the development of new strategies for the rational design of ligands against multidrug-resistant cancer cells.

## 1. Introduction

Since their first description [1], 40 years of research on microtubule targeting agents (MTAs) has expanded our knowledge of biologically potent tubulin-binding compounds. Many studies led to the characterization of new microtubule targeting agent (i.e., microtubule-stabilizing and -destabilizing agents; referred to as MSAs and MDAs, respectively), some of which are routinely used in the clinic and a large number of which are currently under clinical development. In recent years, high resolution structural studies on tubulin-drug complexes have played a key role in understanding the mechanism of action of MTAs and has inspired the development of more efficient drugs [2,3]. MSAs targeting the taxane site of tubulin are, for example, able to change the conformation of the M-loop of β-tubulin. This conformational change is essential to strengthen the lateral contacts between protofilament in microtubules [4], and to revert changes induced by GTP (guanosine triphosphate) hydrolysis at the longitudinal tubulin-tubulin interface along protofilaments [5]. On the other hand, the laulimalide/peloruside class of ligands stabilizes microtubules both by partial structuration of the M-loop and by bridging two adjacent tubulin dimers across protofilaments in microtubules [6]. MDAs binding at the vinblastine- or maytansine site of tubulin act by interfering with longitudinal tubulin-tubulin interactions required for protofilament formation [7,8], whereas those binding at the colchicine site act as “wedges” that prevent the required straightening of tubulin dimers during microtubule formation [9].

Despite the increased number of MTAs, two major problems are still encountered during their use as therapeutics: (1) the development of resistance against the available drugs and (2) the undesirable side effects. One strategy commonly used to overcome non-specific side effects is to exploit antibody-drug conjugates (ADCs) to specifically target cancer cells. This methodology can be further extended by attaching two different MTAs with different modes of action to one and the same antibody in order to decrease the chances of resistance development [10,11,12]. However, the identification and development of novel chemotypes seems the most promising strategy to overcome the problem of resistance development [13,14,15].

Quinolin-6-yloxyacetamides (QAs) were initially identified as fungicides that are highly active against several major phytopathogens [16]. They constitute a chemical class of ligands that acts by inhibiting tubulin polymerization [16]. However, their molecular mechanism of action remains unresolved. Here, we found that QAs have an anti-proliferative effect on human tumor cells. Importantly, QAs are highly effective in inhibiting the proliferation of multidrug-resistant cancer cells that overexpress P-glycoproteins. X-ray crystallography led to the identification of their binding site on tubulin and clarified the molecular mechanism underlying the microtubule destabilization activity of QAs.

## 2. Results

### 2.1. Effect of Quinolin-6-Yloxyacetamides on Cells

**Figure 1:**
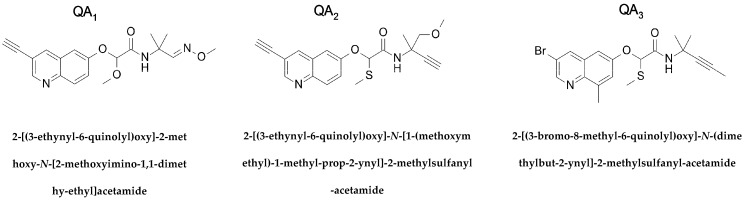
Chemical structures of the quinolin-6-yloxyacetamides compounds used in this study.

QAs act as fungicides by inhibiting tubulin polymerization [16]. In view of previous results, we wanted to evaluate the activity of QA derivatives on tumoural cells. To do so, we assessed the anti-proliferative activity of three different QA derivatives (denoted by QA_1_, QA_2_, and QA_3_; Figure 1) against ovarian and *P*-glycoprotein (PGP)-overexpressing human carcinoma cell lines (A2780 and A2780AD, respectively), lung carcinoma A549, and NIH mouse embryo 3T3 cells using an MTT (3-(4,5-dimethylthiazol-2-yl)-2,5-diphenyltetrazolium bromide)-based assay. The IC_50_ values after a 48-h treatment are summarized in Table 1. We found that all three QA compounds tested show anti-proliferative effects on A2780 and A549 cells in the sub-micromolar range, with QA_1_ and QA_2_ being more potent than QA_3_. Importantly, their growth inhibitory effect remains essentially unchanged in multiple drug-resistant PGP overexpressing A2780AD cells, since their IC_50_ values in both A2780 and A2780AD cells are not statistically different. In comparison to paclitaxel, which displays a resistance index of 916, in A2780AD cells the resistance index towards QAs is less than 3.

**Figure 2:**
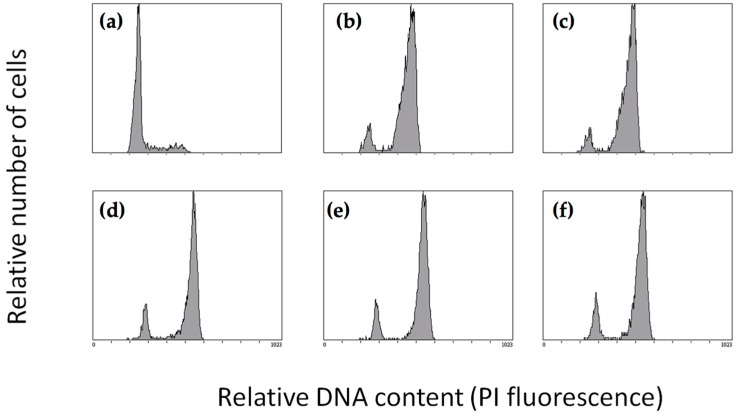
Cell cycle histograms. A549 lung carcinoma cells were incubated for 20 h with either (**a**) DMSO (Dimethylsulfoxyde); (**b**) 250 nM QA_1_; (**c**) 250 nM QA_2_; (**d**) 10 µM QA_3_; (**e**) 100 nM Colchicine; or (**f**) 50 nM Pironetin. The histograms shown correspond to the lowest ligand concentration that induces maximal cell cycle arrest in the G2/M phase, and are generated by counting 5000 cells by flow cytometry.

Given the fact that the compounds were found to have growth inhibitory effects, we continued to investigate their actual cellular mechanism of action in human cells. Since MTAs usually induce G2/M cell cycle arrest, the effect of QAs on the cell cycle was studied for each compound with A549 lung carcinoma cells for 20 h by flow cytometry. Figure 2 shows the DNA histograms in the presence of ligands and controls. The results indicate that all three QAs block cells in the G2/M phase of the cell cycle, indicating an antimitotic mode of action. The Supplementary Table S1 summarizes the percentage of cells in each phase of the cell cycle and at the indicated ligand concentrations.

### 2.2. Effect on Cellular Microtubules

**Figure 3:**
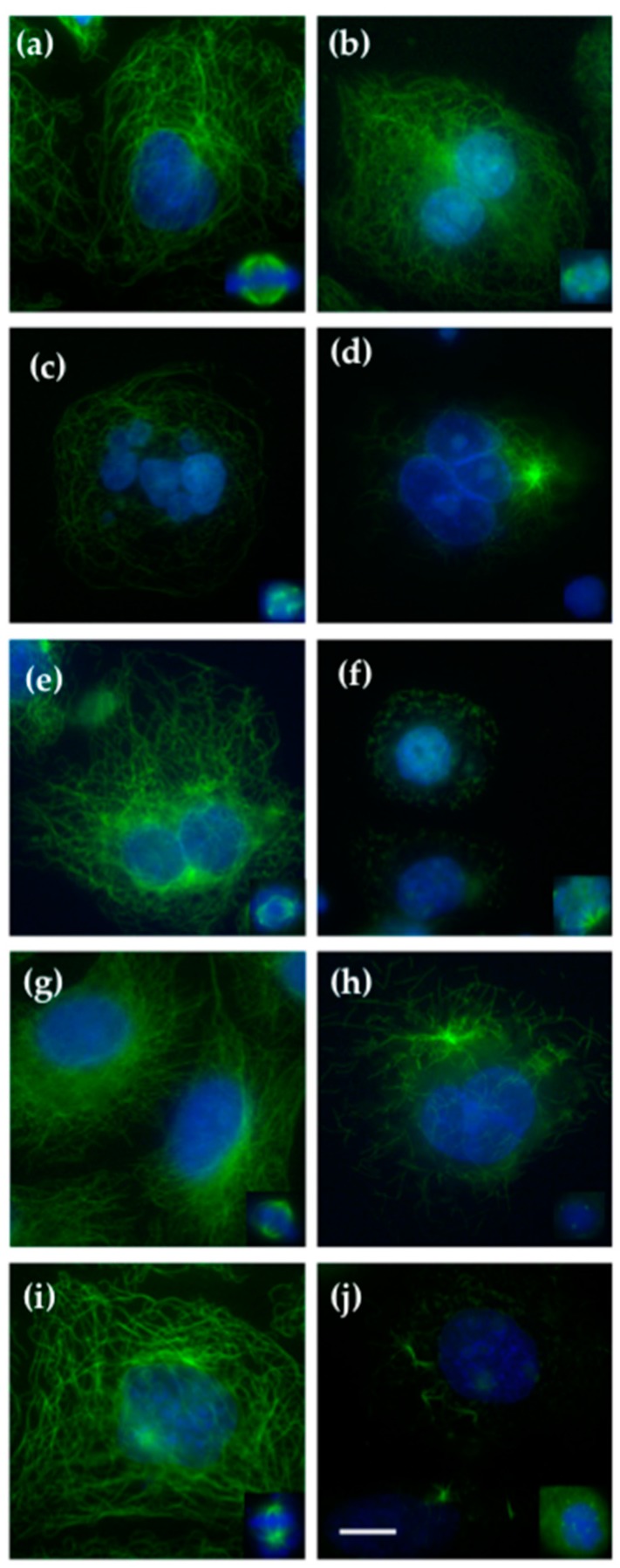
Effect of quinolin-6-yloxyacetamides on the microtubule cytoskeleton network and mitotic spindles. A549 cells were treated either with (**a**) DMSO; (**b**) 100 nM QA_1_; (**c**) 250 nM QA_1_; or (**d**) 0.5–1 µM QA_1_; (**e**) 100 nM QA_2_; (**f**) 0.5–1 µM QA_2_; (**g**) 1 µM QA_3_; (**h**) 3–5 µM QA_3_; (**i**) 10 nM vinblastine; or (**j**) 50 nM vinblastine. Microtubules are stained with α-tubulin antibodies (**green**); DNA was stained with Hoechst 33342 (**blue**). The insets are mitotic spindles from the same preparations. Scale bar, 10 µm. All panels and insets have the same magnification.

To probe the effect of QAs on the microtubule cytoskeleton and on the mitotic spindle, we incubated A549 cells with serial concentrations of compounds for 24 h followed by immunofluorescence analysis. Compared to control cells, the three QA compounds tested exerted a depolymerizing effect that is highlighted by the fragmented microtubule cytoskeleton seen in all treated cells (Figure 3). Although all the compounds of the QA series were effective in disrupting cellular microtubules in the low micromolar range, compounds QA_1_ and QA_2_ were the most potent: 100 nM of either of them induced mitotic aberrations (Figure 3b,e). Complete depolymerization of the interphasic microtubule cytoskeleton was achieved in both cases at 0.5–1 μM of compound (Figure 3d,f). In the case of QA_3_, the microtubules were almost not observable anymore in the presence of 3 μM of the compound (Figure 3h). Furthermore, 10 nM of vinblastine used as a control induced aberrant forms of bipolar spindles (type II spindles) with lagging chromosomes (Figure 3, inset i), a phenotype that was also seen with QA_1_ and QA_2_ at the lowest concentrations tested (100 nM, not shown). Type III and IV spindles were seen with 250 and 1 μM of either QA_1_ and QA_2_, and with either 3 μM of QA_3_ (Figure 3, insets c,d,f,h) or 50 nM of vinblastine. Vinblastine effects on microtubules have been reported previously [17], chromosomes were arranged in a “ball” enclosing one or more star-shaped aggregates of tubulin/microtubules; in some cases, no microtubules could be seen, and with 50 nM vinblastine a complete depolymerization of the microtubule cytoskeleton was observed (Figure 3j).

### 2.3. Effect of Quinolin-6-Yloxyacetamides on Microtubule Formation In Vitro

**Figure 4:**
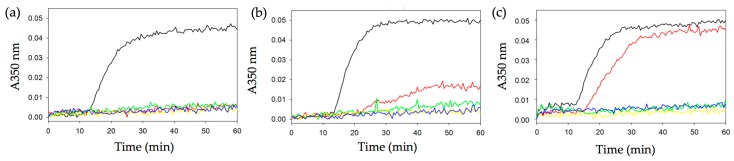
In vitro tubulin polymerization. Turbidity assays for the assembly of 20 μM tubulin at 37 °C in the presence of QA_1_ (**a**), QA_2_ (**b**), and QA_3_ (**c**) followed at 350 nm. **Black** line, DMSO vehicle; **red** line, 2 μM QA; **green** line, 5 μM QA; **yellow** line, 10 μM QA; **blue** line, 25 μM QA.

To gain further insight into the molecular mechanism of action of QAs, we tested their effect on the assembly of microtubules by using a standard tubulin polymerization assay (Figure 4). Briefly, twenty μM of tubulin was incubated in glycerol-assembly buffer (GAB) for 70 min at 37 °C in the presence of different amounts (2–25 μM) of compounds. The degree of tubulin polymerization was then evaluated by monitoring the absorbance at 350 nm. We found that all QAs were strong inhibitors of tubulin polymerization, with almost complete inhibition of tubulin polymerization at 5 μM concentration of compounds. The polymerization inhibition observed for each compound is in agreement with their anti-proliferative activity.

### 2.4. Crystal Structure of the Tubulin-QA_1_ Complex

To understand the binding mode and mechanism of action of QAs on tubulin and microtubules, we solved the crystal structure of αβ-tubulin in complex with the most potent compound, QA_1_, by using the well-established T_2_R-TTL crystallization system [4]. The T_2_R-TTL−QA_1_ complex structure was determined at 2.4 Å resolution (Supplementary Table S2). Unambiguous difference electron density for QA_1_ was observed on both tubulin subunits in the T_2_R-TTL-QA_1_ complex, which led us to model the drug molecule confidently (Supplementary Figure S1a).

**Figure 5:**
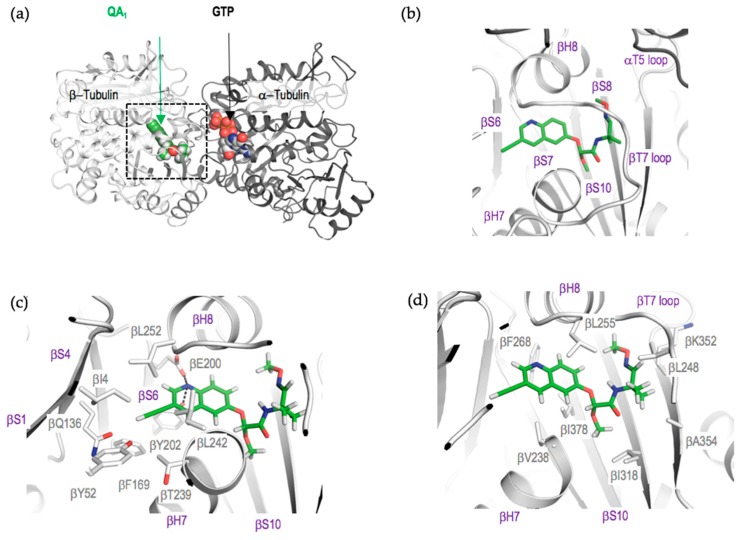
QA_1_ binds at the colchicine site of tubulin. (**a**) Overall view of the tubulin-QA_1_ complex. QA_1_ is displayed as **green** spheres; (**b**) A close up view of the region highlighted with a dashed rectangle in (**a**) and showing the QA_1_ binding site. QA_1_ is displayed as **green** sticks. Major secondary structure elements are labelled; (**c**,**d**) Interacting residues of β-tubulin with QA_1_. Oxygen and nitrogen atoms are displayed in red and blue, respectively.

**Figure 6:**
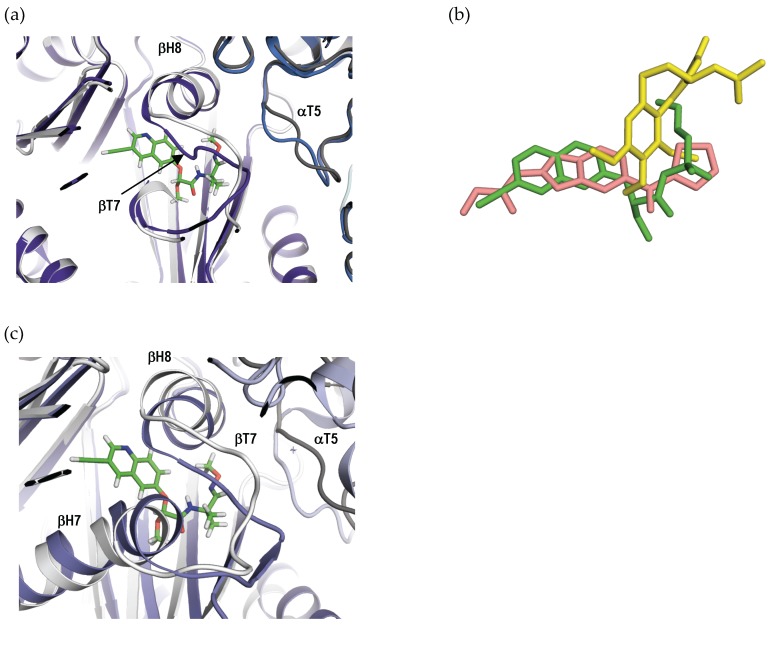
Mechanism of QA_1-_mediated microtubule destabilization. (**a**) Superimposition of tubulin in its QA_1_ bound (**white** ribbons, β-tubulin; **grey** ribbons, α-tubulin) and unliganded (**purple** ribbons, β-tubulin; **blue** ribbons, α-tubulin). The β-tubulin subunits were used for the superimposition. QA_1_ is displayed as **green** sticks; (**b**) Superimposition of QA_1_ (**green** sticks) with colchicine (**yellow** sticks, PDB ID 4O2B) and nocodazole (**pink** sticks, PDB ID 5CA1); (**c**) Superimposition of QA_1_ bound “curved” tubulin (**white**) with “straight” tubulin in microtubules (**purple**). QA_1_ is displayed as **green** sticks. Oxygen and nitrogen atoms are displayed in red and blue, respectively.

QA_1_ binds at the colchicine site on tubulin [18], which is located at the interface between the α- and β-tubulin subunits. It is formed by strands βS1, βS4, βS6, βS7, βS8, βS9, and βS10, loop βT7, and helices βH7 and βH8 of β-tubulin, as well as the loop αT5 of α-tubulin (Figure 5). Similar to nocodazole [19], although the αT5 loop adopts a different conformation upon QA_1_ binding compared to the apo tubulin structure (Figure 6a), we did not observe any direct interactions between QA_1_ and αT5 loop residues. The overall structure of tubulin in the T_2_R-TTL−QA_1_ complex could be readily superimposed with the one obtained in the absence of the ligand (root-mean-square deviation, rmsd, of 0.43 Å over 1904 Cα atoms), suggesting that binding of QA_1_ does not affect the global conformation of the tubulin dimer in the curved state.

QA_1_ binding is mostly established by hydrophobic contacts and a few hydrogen bond interactions with residue side chains of β-tubulin. The ethenyl group at the 3′ position of the quinoline ring of QA_1_ is accommodated in a hydrophobic pocket in β-tubulin delineated by residues βL252, βF169, βL242, βT239, βY52, βQ136, and βI4 (Figure 5c). The nitrogen atom of the quinolone group is involved in hydrogen bond interaction with the side chains of βE200 and βY202. The quinolone ring is accommodated in a hydrophobic cavity formed by the side chains of residues βL242, βL252, βL255 βF268, βI378, and βV238 (Figure 5c,d). The methoxy group substituent in the acetic acid moiety of QA_1_ makes hydrophobic contacts with the side chains of βI318 and βI378 (Figure 5d), whereas the methoxyimino moiety resides in a pocket lined by residues βA354, βL248, and βK352. Most of the β-tubulin residues involved in the interaction with QA_1_ are conserved between mammalian and fungal tubulins, explaining the growth inhibitory effect of QAs against both fungal and mammalian cells (Supplementary Figure S1b).

A comparison of the β-tubulin subunit between the apo-T_2_R-TTL (Protein Data Bank (PDB) ID 4I55) and T_2_R-TTL-QA_1_ shows that the βT7 loop residues βL248 and βN249 of β-tubulin occupies the QA_1_ site in the apo structure. Therefore, to accommodate QA_1_ in its binding site, the βT7 loop has to flip outwards and the αT5 loop of α-tubulin has to change its conformation (Figure 6a). Similar conformational changes have been observed upon binding of other colchicine site ligands to tubulin [3,9,18].

Since QAs form a chemical class of colchicine site binders that are structurally unrelated to colchicine, we superimposed the T_2_R-TTL-colchicine structure onto the T_2_R-TTL-QA_1_ structure. The overall conformation of tubulin remains unchanged between the two structures (rmsd of 0.27 over 1973 CA atoms). Despite sharing the same binding pocket, only the methoxyimino group of QA_1_ overlaps with the colchicine molecule, whereas the quinoline ring of QA_1_ explores regions deep in the β-tubulin subunit remote from colchicine. Interestingly, the quinoline ring of QA_1_ overlaps with nocodazole, which is another colchicine site binder (Figure 6b). QAs are thus a new chemical class of colchicine site, tubulin polymerization inhibitors that bind to an overlapping site in tubulin that is located between the ones of colchicine and nocodazole.

The two tubulin dimers in the T_2_R-TTL structure assume the “curved” conformation characteristic of free tubulin, in contrast with the “straight” tubulin structure that is found in microtubules. To assess whether the binding of QA_1_ is compatible with the straight tubulin conformation present in microtubules, we compared the structures of tubulin in the curved and straight conformational states. We found that in straight tubulin, the QA_1_ binding site is occluded by the βT7 loop of β-tubulin, resulting in severe clashes between the βT7 loop residues with the QA_1_ molecule in the modelled tubulin–QA_1_ structure (Figure 6c). Therefore, QAs cannot bind to preformed microtubules. QAs thus act as microtubule destabilizers by binding to curved tubulin in solution and preventing the curved-to-straight structural transition required for microtubule formation [18]. Together, these data establish QAs as microtubule destabilizers of the colchicine site.

## 3. Discussion

MTAs include a wide variety of small molecules with diverse chemical structures. They interfere with microtubule dynamics, which is an essential process for many cell functions including cell division, angiogenesis, cell migration, and intracellular transport. Because of their anti-proliferative properties, MTAs remain one of the most active areas of research for cancer treatment. Several MTAs are used in cancer chemotherapy and many others are in clinical trials. However, the development of resistance against the available drugs is a major challenge that necessitates the search for and development of MTAs with novel chemical structures and mechanisms of action.

Six distinct ligand binding sites (taxane [20], colchicine [18], vinca [21], laulimalide/peloruside [6], pironetin [22,23], and maytansine [7]) were characterized on tubulin to high resolution using X-ray crystallography. It is well established that ligands belonging to different chemical classes can bind to a common binding site on tubulin and perturb microtubule functions using similar mechanisms of action [19]. Therefore, the search for tubulin inhibitors with novel chemical structures targeting the known drug binding sites on tubulin offers an attractive alternative strategy to overcome multiple drug resistance. However, the rational development of tubulin inhibitors requires the availability of atomic resolution structural information on tubulin-ligand complexes. In this work, we show that QAs constitute a favorable chemical class of MTAs that are highly toxic to multidrug-resistant human cancer cells overexpressing *P*-glycoproteins. We further show that QAs target cellular microtubules, inducing systemic microtubule depolymerization and gross mitotic defects. By solving the high resolution crystal structure of tubulin in complex with QA_1_, we could unambiguously determine that QAs bind to the colchicine site of tubulin. QA_1_ binding prevents the conformational changes in the βT7 loop of β-tubulin accompanying the curved-to-straight tubulin conformational transition required for microtubule formation. Based on our data, we propose that the primary mechanism of action of QAs is to bind to the curved conformational state of tubulin, either when free in solution or when incorporated at the ends of growing microtubules, to lock the protein in a polymerization incompetent state; a similar mechanism of action was postulated for other colchicine site antitubulin ligands [2,24].

Although clinical trials with several colchicine-site ligands are ongoing, none have yet been approved and no colchicine-site ligand is currently in active clinical application, highlighting the need to develop colchicine-site agents. Many structurally diverse ligands are known to target the colchicine site of tubulin and to differentially explore its sub-pockets [3,19]. In this context, QAs with their favorable chemical scaffolds and activity profiles against multi drug resistant (MDR) cancer cells represent attractive colchicine-site ligand candidates. Our structural and biochemical work on QAs presented here may thus offer a favorable basis for the rational design of novel and potent QA derivatives against different types of tumors.

## 4. Materials and Methods

### 4.1. Proteins and Ligands

Purified calf-brain tubulin and chemicals were prepared as described previously [25]. Compounds QA_1–3_ (Figure 1) were synthesized as described [26,27,28]. They were dissolved in D6-DMSO at a concentration of 20 mM and stored at −80 °C. The compounds were analyzed using an Agilent 1100 chromatograph connected to a reverse phase column Zorbax Eclipse XDB-C18 (mobile phase 70% methanol in water for 20 min) coupled to an Agilent 6120 mass spectrometer. All the compounds were found to be more than 95% pure. MS-HPLC analyses revealed the expected molecular weights for all three compounds. Their solubility in water was determined by centrifuging at 110,000× *g*. Then, 50 μM samples were stored in polypropylene or glass tubes in 10 mM sodium phosphate, pH 6.5, 3.4 M glycerol, 1 mM EGTA (ethylene-*bis*(oxyethylenenitrilo)tetraacetic acid), 6 mM MgCl_2_, 0.1 mM GTP. The concentration of each compound in solution was determined spectrophotometrically before and after centrifugation. All the compounds were found to be soluble at a concentration of 50 μM.

### 4.2. Tubulin Polymerization Assay

Polymerization of tubulin in vitro was performed with 100 μL samples and monitored by absorbance at 350 nm (Filter A00019x) in 96-well plates (Falcon, transparent, flat bottom) with an Appliskan plate reader (Thermo Fisher Scientific, Waltham, MA, USA). Subsequently, twenty μM of tubulin in GAB buffer (10 mM sodium phosphate, pH 6.7, 30% glycerol, 1 mM EGTA, 6 mM MgCl_2_, 1 mM GTP) was supplemented with increasing amounts of the compounds up to 25 μM, or with DMSO as a vehicle control. Reactions were monitored for 70 min at 37 °C. Data were exported using the Thermo Scientific SkanIt software of Appliskan (version 2.3, Thermo Scientific, Waltham, MA, USA), and Excel software was used to generate plots. For convenience, data were normalized to the minimum absorbance value of the initial stable plateau.

### 4.3. Cell Culture and Cell Biology Assays

Human A549 non-small lung carcinoma cells and human ovarian carcinomas A2780 and A2780AD (MDR overexpressing *P*-glycoprotein) were cultured as previously described [29]. NIH 3T3 mouse embryo cells were cultured in DMEM (Dulbecco’s modified eagle medium) supplemented with 10% FCS (fetal calf serum), penicillin (100 units/mL) and streptomycin (100 μg/mL). Indirect immunofluorescence, cell cycle analysis, and cytotoxicity assays were performed as described before [30]. The statistical significance of differences in IC_50_ values was evaluated using the *t*-test option implemented in the Sigma Plot 13 software package (version 13, Systat Software, Inc., San Jose, CA, USA).

### 4.4. Complex Reconstitution, Crystallization and Soaking of T_2_R-TTL (Tubulin-RB3-Tubulin Tyrosine Ligase Complex) Crystals with QA_1_

Crystals of T_2_R-TTL were generated as described previously [4,31]. Suitable T_2_R-TTL crystals were incubated for 3 h with reservoir solution supplemented with 5 mM of QA_1_ and 18% glycerol as cryoprotectant. Crystals were flash-cooled in liquid nitrogen.

### 4.5. Data Collection and Structure Solution

A single T_2_R-TTL-QA_1_ crystal was used for the collection of native X-ray diffraction data at 100 K at the X06DA beamline at the Swiss Light Source (Paul Scherrer Institut, Villigen, Switzerland). Data were processed using the XDS software package [32]. The T_2_R-TTL-QA_1_ complex crystallized in space group P2_1_2_1_2_1_ with a single molecule in the asymmetric unit. Structure solution and refinement was performed using the PHENIX software package [33] as described previously [4,31]. Briefly, phases from the T_2_R-TTL complex (PDB ID 4I4T) in the absence of ligands and solvent were used for a structure solution by a few cycles of rigid-body refinement in PHENIX. The model was further refined using multiple cycles of simulated annealing and restraint refinement. The resulting model was improved through the iterative model rebuilding in Coot [34] and refinement in PHENIX. The quality of the structure was assessed with MolProbity [35].

Data collection and refinement statistics are given in Supplementary Table S2. Figures were prepared using PyMOL (The PyMOL Molecular Graphics System, Version 1.4.1. Schrödinger, LLC, (Cambridge, MA, USA)).

## Figures and Tables

**Table 1:** Anti-proliferative effect of quinolin-6-yloxyacetamides in A2780, A2780AD, A549, and 3T3 cells.

Compound	A549	A2780	A2780AD	R/S	3T3
QA_1_	60 ± 2	71 ± 14	141 ± 42	1.9	79 ± 5
QA_2_	44.3 ± 11	104 ± 8	262 ± 85	2.5	125 ± 9
QA_3_	707 ± 23	900 ± 100	800 ± 80	0.9	2400 ± 600
Paclitaxel	3.2 ± 1	1.2 ± 0.1	1100 ± 300	916	ND
Colchicine	55 ± 4	13.6 ± 2	663 ± 23	48	62.2 ± 4

IC_50_ (nM, mean ± standard error) values of the ligands determined in lung carcinoma A549, in ovarian carcinoma A2780 and A2780AD, and mouse embryo NIH 3T3 (3T3) cell lines. The resistance index, R/S, is obtained by dividing the IC_50_ of the resistant A2780AD cell line by that of the parental A2780 cell line. ND, not determined. Values represent the mean ± standard error of three independent experiments.

## Supplementary Materials

Supplementary materials can be found at www.mdpi.com/1422-0067/18/7/1336/s1. Coordinates and structure factors for T2R-TTL-QA_1_ have been deposited in the PDB with the accession code 5O7A.

## Abbreviations

QAs	Quinolin-6-yloxyacetamides
MT	Microtubules
MSA	Microtubule stabilizing agent
MDA	Microtubule destabilizing agent
