# Supplementary material for: Quinolin-6-Yloxyacetamides Are Microtubule Destabilizing Agents That Bind to the Colchicine Site of Tubulin
Source: Int J Mol Sci. 2017 Jun 22;18(7):1336. doi: 10.3390/ijms18071336 (PMC5535829; doi:10.3390/ijms18071336)
Supplement: Supplementary file 1 [file ijms-18-01336-s001.pdf]

**Table S1.** Cell cycle distribution of A549 cells treated with QAs and controls.

| Compound | G0/G1 | S | G2/M |
|---|---|---|---|
| DMSO | 81 | 7 | 12 |
| 250 nM $QA_1$ | 11 | 4 | 85 |
| 250 nM $QA_2$ | 12 | 7 | 81 |
| 250 nM $QA_3$ | 10 | 6 | 84 |
| 100 nM colchicine | 18 | 5 | 77 |
| 50 nM pironetin | 17 | 7 | 76 |

**V**alues represent percentages (%).

**Table S2.** X-ray date collection and refinement statistics.

| | |
|---|---|
| **Data Collection** [a] | |
| Wavelength, Å | 1 |
| Space group | P 21 21 21 |
| Resolution range, Å | 47.98 – 2.495 (2.584 –2.495) |
| Unite cell a, b, c (Å) $\alpha$, $\beta$, $\gamma$ (°) | 104.907 157.474 180.678 90 90 90 |
| No. of observed reflections | 1396075 (122343) |
| No. of unique reflections | 104223 (9893) |
| Mean I/sigma (I) | 13.94 (0.76) |
| R-merge | 0.2069 (3.31) |
| R-meas | 0.2151 (3.451) |
| CC1/2 [b] | 0.998 (0.231) |
| CC * | 0.999 (0.613) |
| **Refinement** | |
| R-work | 0.2041 (0.3525) |
| R-free | 0.2559 (0.3691) |
| Macromolecules | 16643 |
| Ligands | 207 |
| Protein residues | 2089 |
| RMS (bonds) (Å) | 0.011 |
| RMS (angles) (°) | 1.11 |
| Ramachandran favored (%) [c] | 96 |
| Ramachandran outliers (%) [c] | 0.048 |
| **B-factors** | |
| Average B-factor | 72.02 |
| Macromolecules | 72.23 |
| Ligands | 66.22 |
| Solvent | 55.29 |

[a] Highest resolution shell statistics are in parentheses. [b] As defined by Karplus and Diederichs (PMID: 22628654). [c] As defined by MolProbity (PMID: 15215462)

(a)

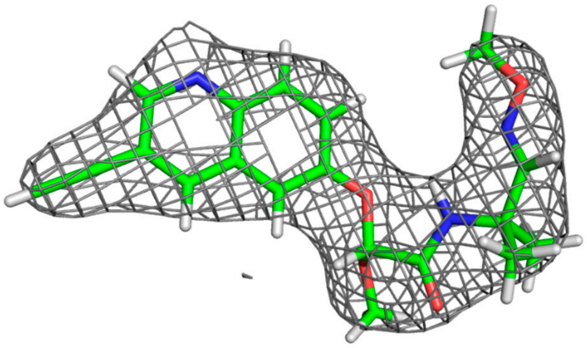

(b)

```
                     1        10         20         30         40         50         60         70         80         90
Bos torous           MREIVHIQAG QCG-NQIGAK FWEVISDEHG IDPTGSYHGD SDLQLERINV YYNEATGNKY VPRAILVDLE PGTMDSVRSG PFGQIFRPDN
Erysiphe necator     MREIVHLQTG QCG-NQIGAA FWQTISGEHG LDGSGVYNGT SDLQLERTNV YFNEASGNKY VPRAVLVDLE PGTMDAVRAG PFGQLFRPDN
Pythium ultimum      MRELVHIQGG QCG-NQIGAK FWEVISDEHG VDPTGSYHGD SDLQLERINV YYNEATGGRY VPRAILMDLE PGTMDSVRAG PFGQLFRPDN
Zymoseptoria tritici MREIVHLQTG QCVSNQIGAA FWQTISGEHG LDGSGVYNGT SDLQLERMNV YFNEASGNKY VPRAVLVDLE PGTMDAVRAG PFGQLFRPDN
Phytophthora infestans MRELVHIQGG QCG-NQIGAK FWEVISDEHG VDPTGSYHGD SDLQLERINV YYNEATGGRY VPRAILMDLE PGTMDSVRAG PYGQLFRPDN

                            100        110        120        130        140        150        160        170        180
Bos torous           FVFGQSGAGN NWAKGHYTEG AELVDSVLDV VRKESESCDC LQGFQLTHSL GGGTGSGMGT LLISKIREEY PDRIMNTFSV MPSPKVSDTV
Erysiphe necator     FVFGQSGAGN NWAKGHYTEG AELVDQVLDV VRREAEGCDC LQGFQITHSL GGGTGAGMGT LLISKIREEF PDRMMATFSV VPSPKVSDTV
Pythium ultimum      FVFGQPGAGN NWAKGHYTEG AELIDSVLDV ARKEAESCDC LQGFQITHSL GGGTGSGMGT LLISKIREEY PDRIMCTYSV CPSPKVSDTV
Zymoseptoria tritici FVFGQSGAGN NWAKGHYTEG AELVDQVLDV VRREAEGCDC LQGFQITHSL GGGTGAGMGT LLISKIREEF PDRMMATFSV MPSPKVSDTV
Phytophthora infestans FVFGQTGAGN NWAKGHYTEG AELIDSVLDV VRKEAESCDC LQGFQITHSL GGGTGSGMGT LLISKIREEY PDRIMCTYSV CPSPKVSDTV

                            190        200        210        220        230        240        250        260        270
Bos torous           VEPYNATLSV HQLVENTDET YCIDNEALYD ICFRTLKLTT PTYGDLNHLV SATMSGVTTC LRFPGQLNAD LRKLAVNMVP FPRLHFFMPG
Erysiphe necator     VEPYNATLSV HQLVENSDET FCIDNEALYE ICMRTLKLSN PSYGDLNHLV SAVMSGVTTC LRFPGQLNSD LRKLAVNMVP FPRLHFFMVG
Pythium ultimum      VEPYNATLSV HQLVENADEV MCLDNEALYD ICFRTLKLTT PTYGDLNHLV CAAMSGITTC LRFPGQLNSD LRKLAVNLIP FPRLHFFMIG
Zymoseptoria tritici VEPYNATLSV HQLVENSDET FCIDNEALYD ICMRTLKLNN PSYGDLNHLV SAVMSGVTTC LRFPGQLNSD LRKLAVNMVP FPRLHFFMVG
Phytophthora infestans VEPYNATLSV HQLVENADEV MCLDNEALYD ICFRTLKLTT PTYGDLNHLV CAAMSGITTC LRFPGQLNSD LRKLAVNLIP FPRLHFFMIG

                            280        290        300        310        320        330        340        350        360
Bos torous           FAPLTSRGSQ QYRALTVPEL TQQMFDSKNM MAACDPRHGR YLTVAAIFRG RMSMKEVDEQ MLNVQNKNSS YFVEWIPNNV KTAVCDIPPR
Erysiphe necator     FAPLTSRGAH SFRAVTVPEL TQQMYDPKNM MAASDFRNGR YLTCSAIFRG KVSMKEVEDQ MRNVQNKNSA YFVEWIPNNV QTALCSIPPR
Pythium ultimum      FAPLTSRGSQ QYRALTVPEL TQQQFDAKNM MCAADPRHGR YLTAACMFRG RMSTKEVDEQ MLNVQNKNSS YFVEWIPNNI KASVCDIPPK
Zymoseptoria tritici FAPLTSRGAH SFRAVTVPEL TQQIFDPKNM MAASDFRNGR YLTCSAIYRG KVSMKEVEDQ IRNVQNKNTA YFVEWIPNNV QTALCSIPPR
Phytophthora infestans FAPLTSRGSQ QYRALTVPEL TQQQFDAKNM MCAADPRHGR YLTAACMFRG RMSTKEVDEQ MLNVQNKNSS YFVEWIPNNI KASVCDIPPK

                            370        380        390        400        410        420        430        440      448
Bos torous           GLKMSATFIG NSTAIQELFK RISEQFTAMF RRKAFLHWYT GEGMDEMEFT EAESNMNDLV SEYQQYQDAT ADEQGE-FEE EEGEDEA
Erysiphe necator     GLKMSSTFVG NSTSIQELFK RVGDQFTAMF RRKAFLHWYT GEGMDEMEFT EAESNMNDLV HEYQQYQDAS ISEGEEDYEE EPQVENEE
Pythium ultimum      GLKMSTTFVG NSTAIQEMFK RVSEQFTAMF RRKAFLHWYT GEGMDEMEFT EAESNMNDLV SEYQQYQDAT AEEEGE-FDE DEEMDEMM
Zymoseptoria tritici GLKMSSTFVG NSTSIQELFK RVGDQFSAMF RRKAFLHWYT GEGMDEMEFT EAESNMNDLV SEYQQYQEAS VSDAEEEYDE EAPLEGEE
Phytophthora infestans GLKMSTTFIG NSTAIQEMFK RVSEQFTAMF RRKAFLHWYT GEGMDEMEFT EAESNMNDLV SEYQQYQDAT AEEEGE-FDE DEEMDEMM
```

**Figure S1.** Electron density map and multiple sequence alignment. (**a**) Electron density map showing fitting of QA1 in the electron density; (**b**) Multiple sequence alignment of mammalian beta-tubulin with beta-tubulin from important fungal phytopathogens. Amino acid residues involved in QA1 are highlighted by black stars.
